# Sparking Nanometal Oxides for Modifying Surface Properties of Thermoplastic Starch/Carboxymethyl Cellulose Blends

**DOI:** 10.3390/polym18202571

**Published:** 2026-10-19

**Authors:** Araya Kodsangma, Itchaya Thinnakorn, Pornchai Rachtanapun, Winita Punyodom, Pensak Jantrawut, Patnarin Worajittiphon, Nuttapol Tanadchangsaeng, Kittisak Jantanasakulwong

**Affiliations:** 1 Nanoscience and Nanotechnology (International Program/Interdisciplinary), Faculty of Science, Chiang Mai University, Chiang Mai 50200, Thailand; mymild_araya@yahoo.com (A.K.); pornchai.r@cmu.ac.th (P.R.); winita.punyodom@cmu.ac.th (W.P.); patnarin.w@cmu.ac.th (P.W.); 2 Faculty of Agro-Industry, Chiang Mai University, Chiang Mai 50100, Thailand; 3 Center of Excellence in Materials Science and Technology, Faculty of Science, Chiang Mai University, Chiang Mai 50200, Thailand; pensak.amuamu@gmail.com; 4 Center of Excellence in Agro Bio-Circular-Green Industry (Agro BCG), Chiang Mai University, Chiang Mai 50100, Thailand; 5 Department of Chemistry, Faculty of Science, Chiang Mai University, Chiang Mai 50200, Thailand; 6 Department of Pharmaceutical Sciences, Faculty of Pharmacy, Chiang Mai University, Muang, Chiang Mai 50200, Thailand; 7 College of Biomedical Engineering, Rangsit University, Pathumthani 12000, Thailand; nuttapol.t@rsu.ac.th

**Keywords:** thermoplastic starch, sparking process, nanoparticle, sustainable agriculture

## Abstract

A thermoplastic starch (TPS) was developed by blending cassava starch in a 70/30% wt/wt ratio. Carboxymethyl cellulose (CMC) 5% wt/wt was added during TPS solution mixing at 80 °C. Sparking technology was applied to deposit nanometal particles onto polymer surfaces. Nanometal oxide particles of Ag, Zn, and Cu were sparked on the surface of TPS and TPS/CMC samples to investigate the effects of the metal type and polymer surface chemistry on the resulting material properties. The surface morphology, element distribution, mechanical properties, color characteristics, wettability, and thermal behavior were evaluated. The tensile strength of TPS/CMC increased from 2.2 MPa to 5.0 MPa with Ag sparking, while the contact angle of TPS/CMC increased from 41.3° to 91.6°, 89.3°, and 95.1° after Ag, Zn, and Cu sparking, respectively. SEM–EDS confirmed the deposition and distribution of the nanometal types on the polymer surfaces. The sparked TPS/CMC samples exhibited higher contact angles than the sparked TPS sample due to the strong interactions between the deposited nanometal particles and –COOH functional groups of CMC. The thermal analysis showed that the deposited metal type influenced the degradation behavior of the TPS and TPS/CMC samples. Sparking technology provided effective surface modification via depositing nanometal particles onto TPS/CMC, resulting in uniform particle distribution and enhanced mechanical and surface wettability properties, with potential applications in the sustainable packaging industry.

## 1. Introduction

Thermoplastic starch (TPS) has attracted significant attention as a sustainable alternative to petroleum-based plastics due to its biodegradability, non-toxicity, and renewability [1,2]. Starch is an abundant polysaccharide that can be sourced from cassava, corn, rice, and mung beans, offering a cost-effective and renewable base material for bioplastic production. Starch possesses a semi-crystalline structure that restricts its thermoplastic behavior. To convert starch into TPS, a plasticizer can be incorporated to weaken hydrogen bonding between starch chains, thereby increasing chain mobility and flexibility [3,4]. Additives are often incorporated to promote the transformation of starch into an amorphous structure, which enables thermal processing without degradation [5].

TPS can be produced with plasticizers such as water, glycerol, or sorbitol to achieve suitable processability and elasticity [6]. During extrusion or melt-mixing, heat and shear forces disrupt starch granules, while the plasticizer hinders recrystallization and stabilizes the amorphous network [7]. Consequently, TPS can be processed using conventional polymer-processing equipment, behaving similarly to petroleum-based plastics [3,8].

The inherent hydrophilicity and brittleness of TPS often limit its mechanical strength and water resistance. Compatibilizers and reinforcing agents have been introduced to improve interfacial adhesion and overall performance, such as glycerol, sorbitol, gelatin, and chitosan enhance flexibility and cohesion between polymer chains [9,10]. Other additives, including chitosan (CTS), chlorhexidine gluconate (CHG), and carboxymethyl cellulose (CMC), have been reported to improve the mechanical, morphological, water-resistant, and thermal properties of TPS composites [3,11,12,13].

Due to its hydrophilic and water-soluble characteristics, CMC has been extensively used as a thickener, stabilizer, and film-forming agent, improving the rheological and mechanical performance of polymer systems. CMC is used as a processing aid in numerous industrial sectors such as the cosmetic, food, pharmaceutical, paper, and textile sectors, where it enhances texture, viscosity, and moisture retention [14]. Moreover, CMC’s renewable and environmentally friendly attributes have led to its growing use in bio-based composites and green polymer development, contributing to improved sustainability of plastic materials.

The application of nanotechnology enhances the performance of biodegradable polymers. Nanomaterials with particle sizes in the 1–100 nm range possess a high surface-area-to-volume ratio and unique surface reactivity, which can improve mechanical, thermal, and antimicrobial properties. Nanometal oxide particles can be produced by sparking nanoparticle technology. This technology uses high-voltage electrical sparks that cause tiny particles of the metal to break off and oxidize, forming nanometal oxide particles [15,16]. Titanium dioxide (TiO_2_), silver oxide (Ag_2_O), and zinc oxide (ZnO) have been innovated for polymer engineering and biomedical applications [17,18,19,20,21]. Nanometal oxide particles produced by sparking technology have been shown to improve strength, stability, and functionality. Solid-state NMR can provide further molecular-level insights into metal–polymer interactions. However, conventional nanoparticle incorporation into biodegradable polymers often results in bulk blending, which induces agglomeration and limits surface-specific modification. Limited studies have focused on the use of sparking technology for direct surface modification with nanometal particles. The relationship between polymer functional groups, metal species, surface deposition, and the resulting mechanical, wettability, and thermal properties remains insufficiently understood. Unlike the conventional bulk blending of metal-containing particles into TPS/CMC composites, this study employed sparking-based surface depositions to modify the film surface. This approach focuses on improving surface and mechanical properties without intentionally incorporating particles throughout the polymer matrix.

Therefore, the present study aimed to develop and characterize a novel TPS/CMC blend with nanometal oxide particles produced using sparking technology. Cassava starch was used as the primary polymer matrix, while CMC was used due to the remaining –COOH functional groups in the main chain structure. Strong ionic interactions between –COOH functional groups of CMC and nanometal particles were expected to enhance interfacial adhesion. Nanometal oxide particles were deposited using sparking technology onto the TPS/CMC surface to improve surface morphological, mechanical, and surface wettability properties. Silver (Ag), zinc (Zn), and copper (Cu) were selected for sparking treatment because of their distinct physicochemical properties and potential functional benefits. Their use as electrode materials allowed the effects of different metal types on the properties of TPS/CMC samples to be investigated.

## 2. Materials and Methods

### 2.1. Materials

Cassava starch (Dragon Fish brand; amylose/amylopectin ratio of 17%/83%, moisture content of 11% by total weight, CAS number 9005-25-8, and molecular weight of 1.34 × 10^8^ g/mol) was obtained from Tong Chan Registered Ordinary Partnership, Bangkok, Thailand. Carboxymethyl cellulose (CMC), CAS number 9004-32-4 Double Swallow brand with DS 0.75–0.95, was purchased from Yok Inter Trade (Chiang Mai) Co., Ltd., Thailand. Glycerol (CAS number 56-81-5) was supplied by Union Science Co., Ltd., Chiang Mai, Thailand. Ag (CAS number 7440-22-4), Zn (CAS number 7440-66-6), and Cu (CAS number 7440-50-8) wires with a diameter of 0.5 mm were purchased from Advent Research Materials Co., Ltd. (Oxford, UK).

### 2.2. Preparation of Samples

**Figure 1:**
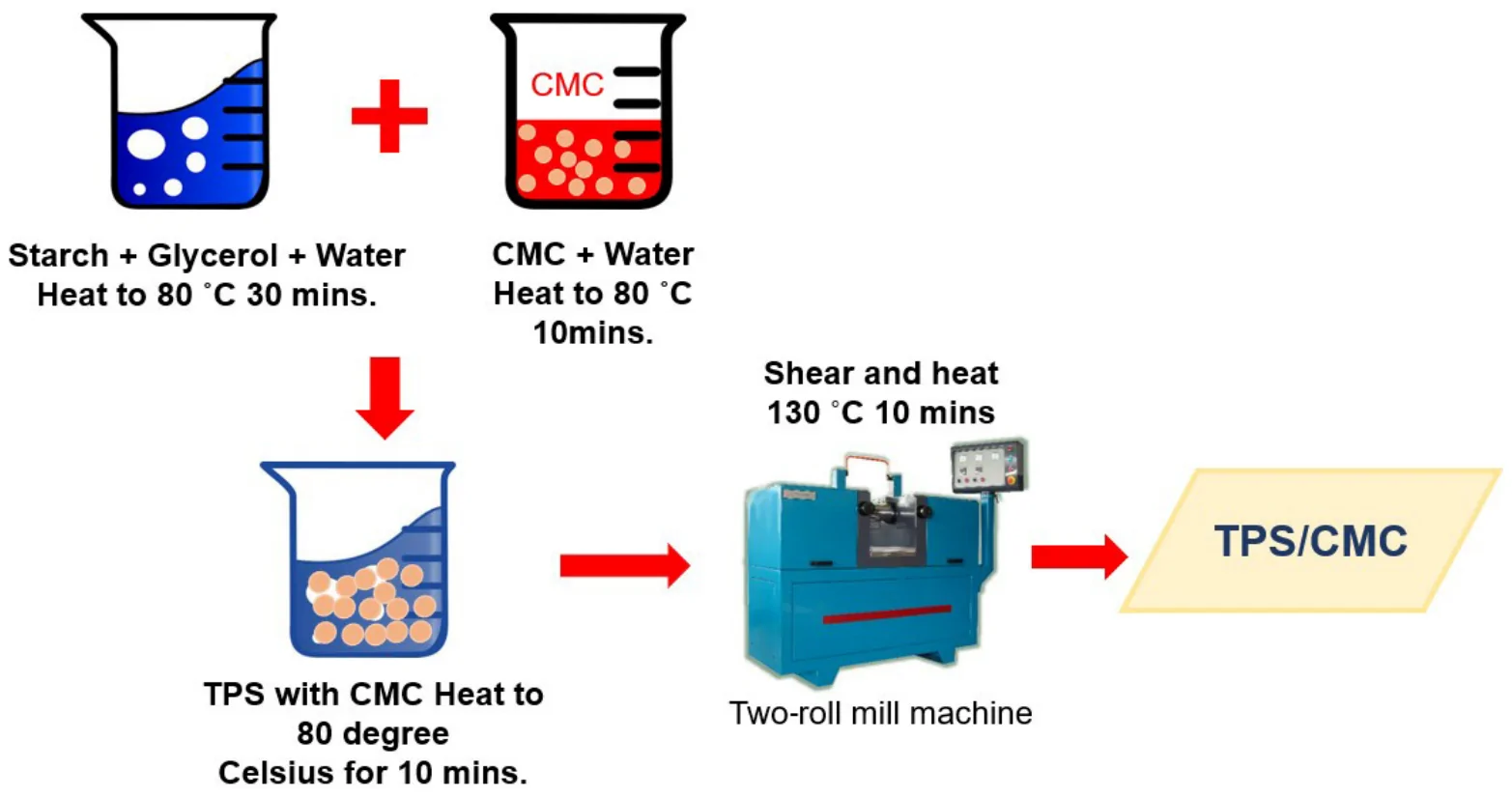
TPS/CMC sample preparation.

First, TPS was prepared by blending cassava starch with glycerol (70/30 wt%) and 200 mL of distilled water in a beaker at 80 °C for 30 min. CMC was incorporated into the preprepared TPS at a TPS/CMC ratio of 95/5 wt%, and mixing was continued for 10 min (Figure 1). The mixture was placed in a heat incubator (Hot Oven; 1370FX-2E; Sheldom Manufacturing Company, OR, USA) at 60 °C for 24 h. The TPS and TPS/CMC samples were prepared using a two-roll mill at 130 °C for 10 min and compressed by compression molding with a hot press (Model 4122–436; Carver, CA, USA) at 130 °C under 7000 psi for 5 min [11]. The untreated TPS/CMC sample was used as the control and was prepared using the same molding process, except that it was not subjected to sparking treatment. Silver (Ag), zinc (Zn), and copper (Cu) wires with a diameter of 0.5 mm were used for the sparking process. The compositions of TPS, CMC, and sparking nanometal oxides are presented in Table 1.

### 2.3. Sparking Method

The sparking process used metal wires (Ag, Zn, or Cu) with two tips (20 mm in length), which were connected to the anode and cathode of the sparking machine. The size of samples was 5 × 5 cm, with a thickness of 1 mm. Sparking was performed on one side of each sample, converting an area of 25 cm^2^. The sparking head remained stationary. The wire tips were located 2 mm above the TPS and TPS/CMC samples, with a 1 mm gap between the anode and cathode. Sparking was conducted at an applied voltage of 3 kV and a current of 3 mA. The sparking process was performed for a total duration of 30 min [22].

### 2.4. Fourier-Transform Infrared (FTIR) Spectroscopy

Fourier-transform infrared (FTIR) spectroscopy was conducted using a Nicolet 6700 spectrometer (Thermo Scientific, Waltham, MA, USA) to investigate the molecular interactions among starch, glycerol, TPS, CMC, TPS/CMC, TPS/Ag, TPS/CMC/Ag, TPS/Zn, TPS/CMC/Zn, TPS/Cu, and TPS/CMC/Cu. Thin-film samples were prepared by compression molding at 130 °C for 5 min. The FTIR spectra were collected within the wavenumber range of 4000–500 cm^−1^ at a resolution of 4 cm^−1^.

### 2.5. Tensile Properties

The specimen dimension was 5 × 30 × 1 mm in width, length, and thickness, respectively, and the specimens were compressed by hot-compression molding at 130 °C under 7000 psi for 5 min. The tensile properties were determined using a universal testing machine (Model H1KS, Hounfield Test Equipment, Middlesbrough, UK). The samples were tested with a gauge length of 10 mm and at a test speed of 10 mm/min. The samples were conditioned at 25 °C and 54% relative humidity for 24 h, and the maximum tensile force and elongation at break were subsequently recorded. Stress–strain curves were obtained from the tensile test data to characterize the mechanical behavior of the specimens. Tensile strength was calculated as the maximum tensile force divided by the initial cross-section area of the specimen, while elongation at break was calculated from the extension at failure relative to the initial gauge length. Each condition was tested using five replicates.

### 2.6. Scanning Electron Microscopy (SEM)

The samples were prepared as sheets by compression molding at 130 °C for 5 min with a length, width, and thickness of 10 mm, 10 mm, and 0.2 mm, respectively. The sample surface was coated with a thin layer of gold and examined under an accelerating voltage of 15 kV. Morphological images were captured using a scanning electron microscope (JSM-IT300LV model, Tokyo, Japan).

### 2.7. Delta E Color

Delta E is a quantitative measure of color differences perceived by the human eye. A Delta E Colorimeter (WR108 Precise Color Reader model, Shenzhen Optoelectronic Technology Co., Ltd., Shenzhen, China) was used to observe the color changes of the samples. The samples were prepared as sheets via compression molding at 130 °C for 5 min, with 5 replicates tested for each condition.

### 2.8. Contact Angle

The specimens were 20 mm in length and 10 mm in width and were tested using a drop shape analyzer (DSA30B, KRUSS, Hamburg, Germany). Water droplets were applied to the sample surfaces at intervals ranging from 1 to 10 min. The measurements were conducted on the top side of the samples using a droplet volume of 10 µL. The contact angle was recorded every 1 min for 10 min, with 5 independent measurements performed for each sample. The contact angle of each droplet and the corresponding images were recorded to analyze the surface wettability of the samples.

### 2.9. Thermogravimetric Analysis (TGA)

A thermogravimetric analyzer (STARe-system TGA/DSC3+, Mettler-Toledo Co., Ltd., Greifensee, Switzerland) was used to evaluate the thermal stability of the materials and determine the fraction of volatile components by monitoring weight changes during heating at a constant rate. TGA measurements were conducted under a nitrogen atmosphere. Samples weighing 10–15 mg were heated from 25 to 500 °C at a rate of 15 °C/min.

### 2.10. Statistical Analysis

Statistical analysis of the experimental data was conducted using one-way ANOVA with SPSS software version 17. To identify significant differences among the sample means, Duncan’s multiple range test was applied, at a significance level of *p* < 0.05.

## 3. Results

### 3.1. Reaction Mechanisms

**Figure 2:**
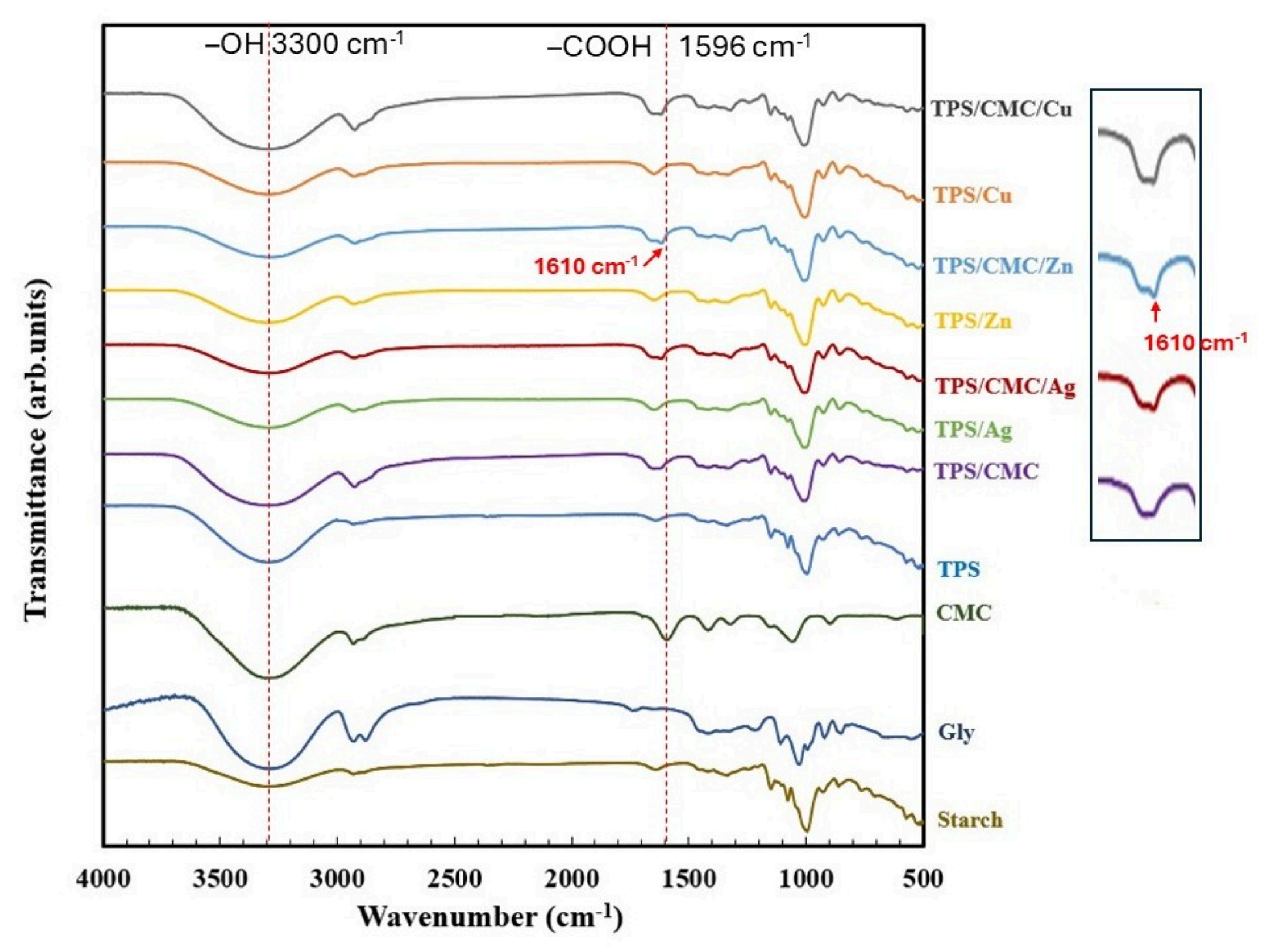
FTIR spectra of starch, glycerol, CMC, TPS, TPS/CMC, TPS/Ag, TPS/CMC/Ag, TPS/Zn, TPS/CMC/Zn, TPS/Cu, and TPS/CMC/Cu.

**Figure 3:**
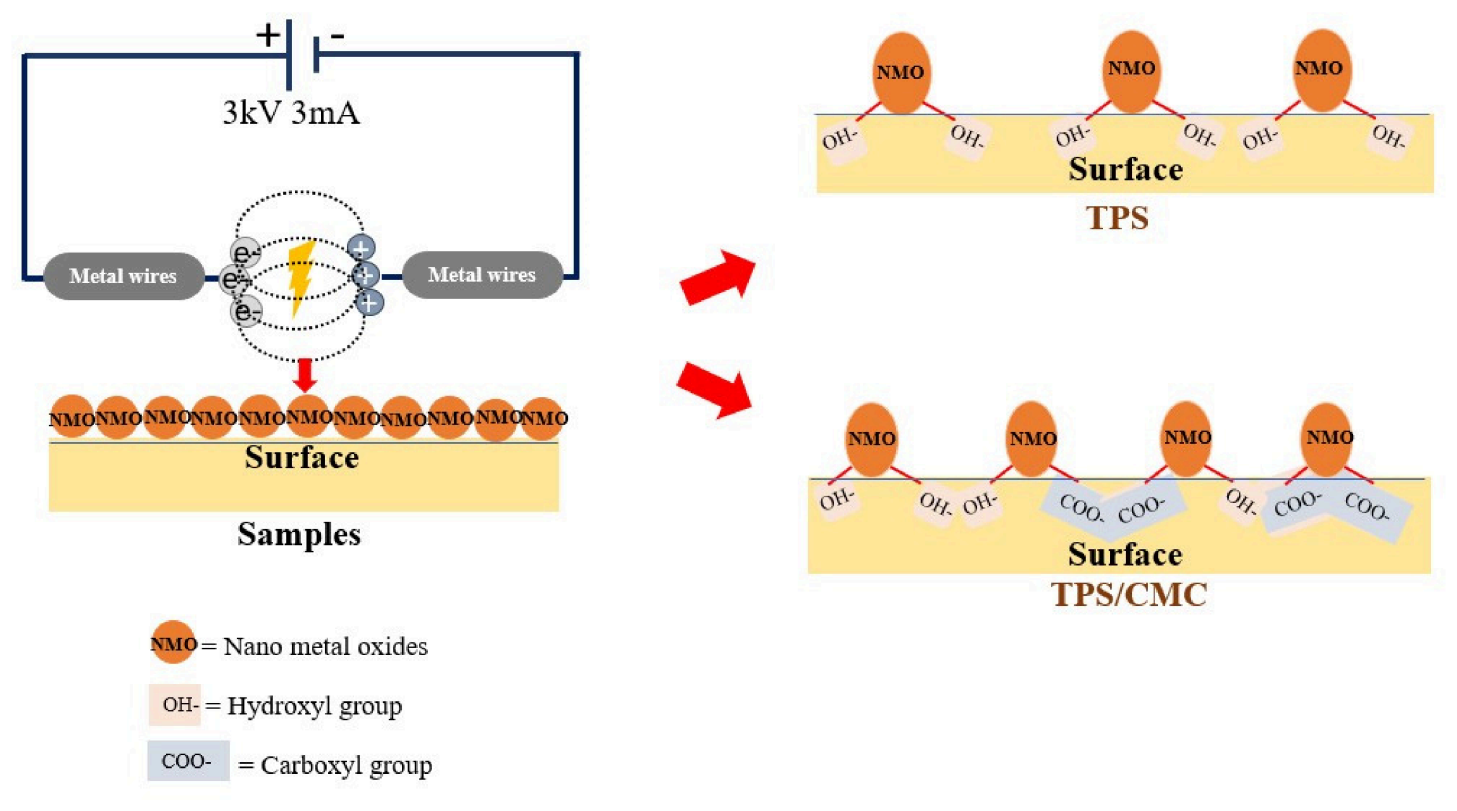
Schematic image of sparking surface process and interaction between nanometal particles with –OH and –COOH groups of samples.

The FTIR vibration peaks of the surfaces of starch, glycerol, CMC, TPS, TPS/CMC, and TPS and TPS/CMC samples sparked with nanometal oxides using sparking technology (TPS/Ag, TPS/Cu, TPS/Zn, TPS/CMC/Ag, TPS/CMC/Cu, TPS/CMC/Zn) were observed at various wavelengths from 500 to 4000 cm^−1^ (Figure 2). TPS exhibited peaks of O–H stretching (3300 cm^−1^), C–H stretching (2920 cm^−1^), bending vibration of bound water (1650 cm^−1^), C–H bending (1423 cm^−1^), and C–O vibrations (1022 and 920 cm^−1^) [3,23,24]. CMC presented peaks of O–H stretching (3290 cm^−1^) [25], asymmetric and symmetric –COO^−^ stretching (1596 and 1410 cm^−1^, respectively) and C–O stretching (1050 cm^−1^) [26]. The FTIR spectrum of the combination of TPS/CMC was between those of TPS and CMC. The peak intensity at 3300 cm^−1^ of the –OH groups of TPS decreased with CMC blending due to the interaction between the –OH groups of TPS and the –COOH groups of CMC. The peak intensity at 3300 cm^−1^ of the –OH groups of TPS and TPS/CMC samples also decreased with Ag, Zn, and Cu nanometal sparking owing to the interaction between the –OH groups of TPS or CMC with nanometal particles (Figure 3). The –COOH group peak of CMC shifted from 1596 to 1610 cm^−1^ due to the interactions of TPS/CMC. The peak intensity at 1610 cm^−1^ of TPS/CMC sparked with Ag, Zn, and Cu was higher compared to TPS/CMC, which indicated a new interaction between the –COOH groups of CMC and the nanometal particles on the sparked surfaces (Figure 2). The interactions between the nanometal particles on the sparked surfaces and –COOH groups were stronger than those with –OH groups [27,28,29]. The decreased O–H stretching (3300 cm^−1^) and the increased intensity of the carboxyl intensity ratio (Table 2) after Ag, Zn, and Cu sparking indicated changes in the molecular interactions at the TPS/CMC surface. As illustrated in Figure 2, the deposited metal-containing species interacted with the –OH groups of TPS and –COOH groups of CMC, promoting interfacial adhesion between the deposited particles and polymer surface. These FTIR changes provided evidence of the proposed interfacial interaction mechanism. The O atoms of –COOH groups interact via asymmetric stretching with Ag, Cu, and Zn ions, forming ionic bond interactions between the deprotonated carboxylate groups of CMC and the nanometal oxides on the surface of the samples [30,31,32,33,34,35], which improved the morphology and mechanical properties of the TPS and TPS/CMC composite nanometal oxides produced via sparking technology.

### 3.2. Tensile Strength

**Figure 4:**
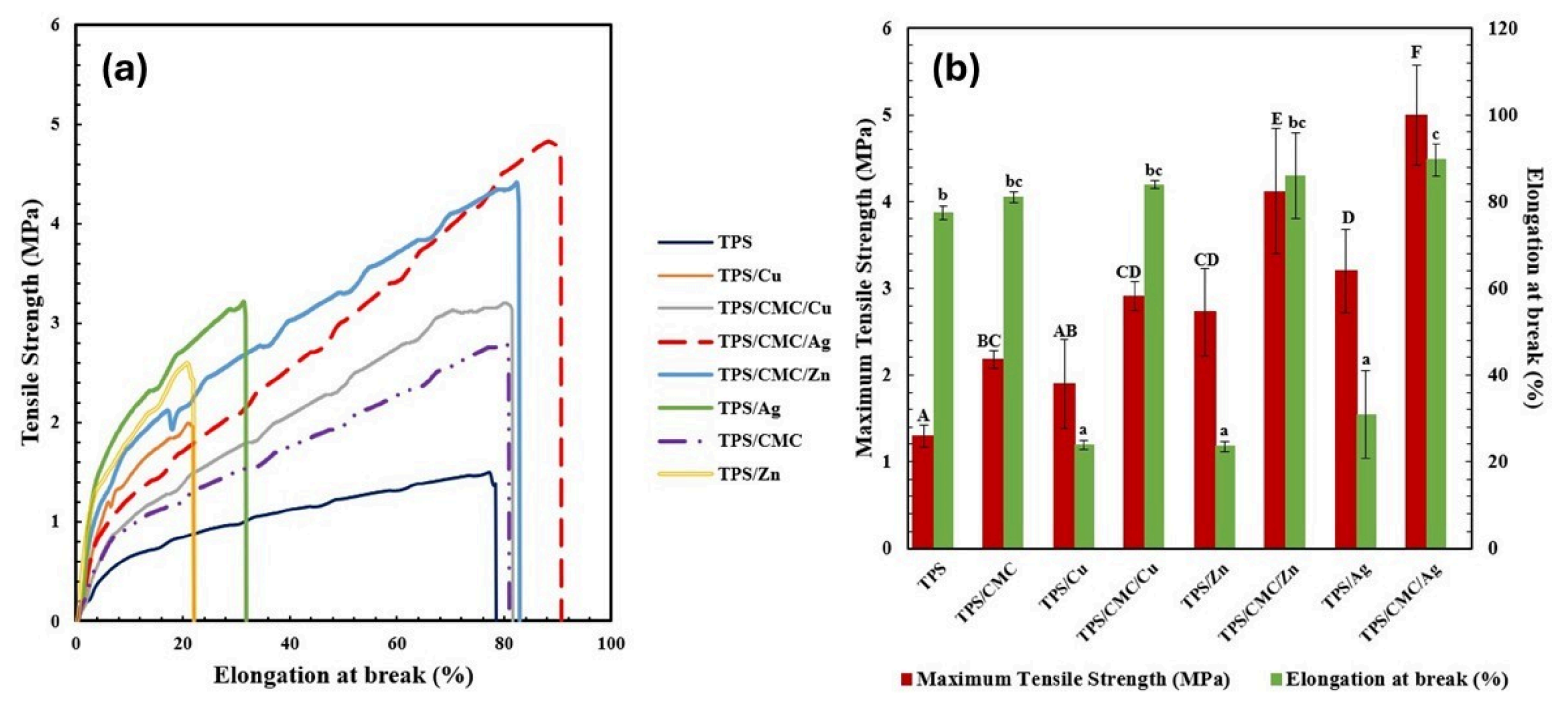
Tensile properties: (**a**) stress–strain curves and (**b**) tensile strength and percent of elongation at break of TPS, TPS/CMC, TPS/Cu, TPS/CMC/Cu, TPS/Zn, TPS/CMC/Zn, TPS/Ag, and TPS/CMC/Ag samples. Data are expressed as mean ± standard deviation. Different letters denote significant differences between samples (*p* < 0.05), where lowercase letters (a–c) indicate differences in elongation at break, and uppercase letters (A–F) indicate differences in tensile strength.

Figure 4 shows the tensile strength and elongation at break of the TPS, TPS/CMC, and TPS and TPS/CMC samples with surfaces sparked with nanometal oxides using sparking technology (TPS/Ag, TPS/Cu, TPS/Zn, TPS/CMC/Ag, TPS/CMC/Cu, TPS/CMC/Zn). TPS showed a tensile strength of 1 MPa and an elongation break at 78%, while the tensile strength of TPS/CMC increased to 2.2 MPa due to the mechanical properties of CMC. The TPS/Ag, TPS/Cu, and TPS/Zn samples showed tensile strengths of 3.2, 1.8 and 2.6 MPa, respectively, while the elongation at break of the TPS/Ag, TPS/Cu, TPS/Zn samples was 21%, 21%, and 30%, respectively. The tensile strength of TPS/CMC/Ag, TPS/CMC/Cu, and TPS/CMC/Zn was 5 MPa, 3 MPa and 4.2 MPa, respectively, while the elongation at break of TPS/CMC/Ag, TPS/CMC/Cu, and TPS/CMC/Zn was 90%, 84%, and 84%, respectively. The increases in the tensile strength and elongation at break of the TPS/CMC/nanoparticle samples indicated ionic bonding interactions between the nanoparticles on the TPS/CMC surface through the –COOH groups of CMC and the polar groups of the nanoparticles [11,30]. Although the deposition of metal oxide nanoparticles on the TPS surface improved the mechanical performance, the enhancement was less than that of the TPS/CMC sample owing to the ionic interactions between the –COOH groups of CMC and the nanoparticles. The nanoparticles forming ionic interactions acted as physical crosslinking points on the TPS/CMC surface, which improved the mechanical properties of the samples [11,31,36,37].

### 3.3. Morphology

**Figure 5:**
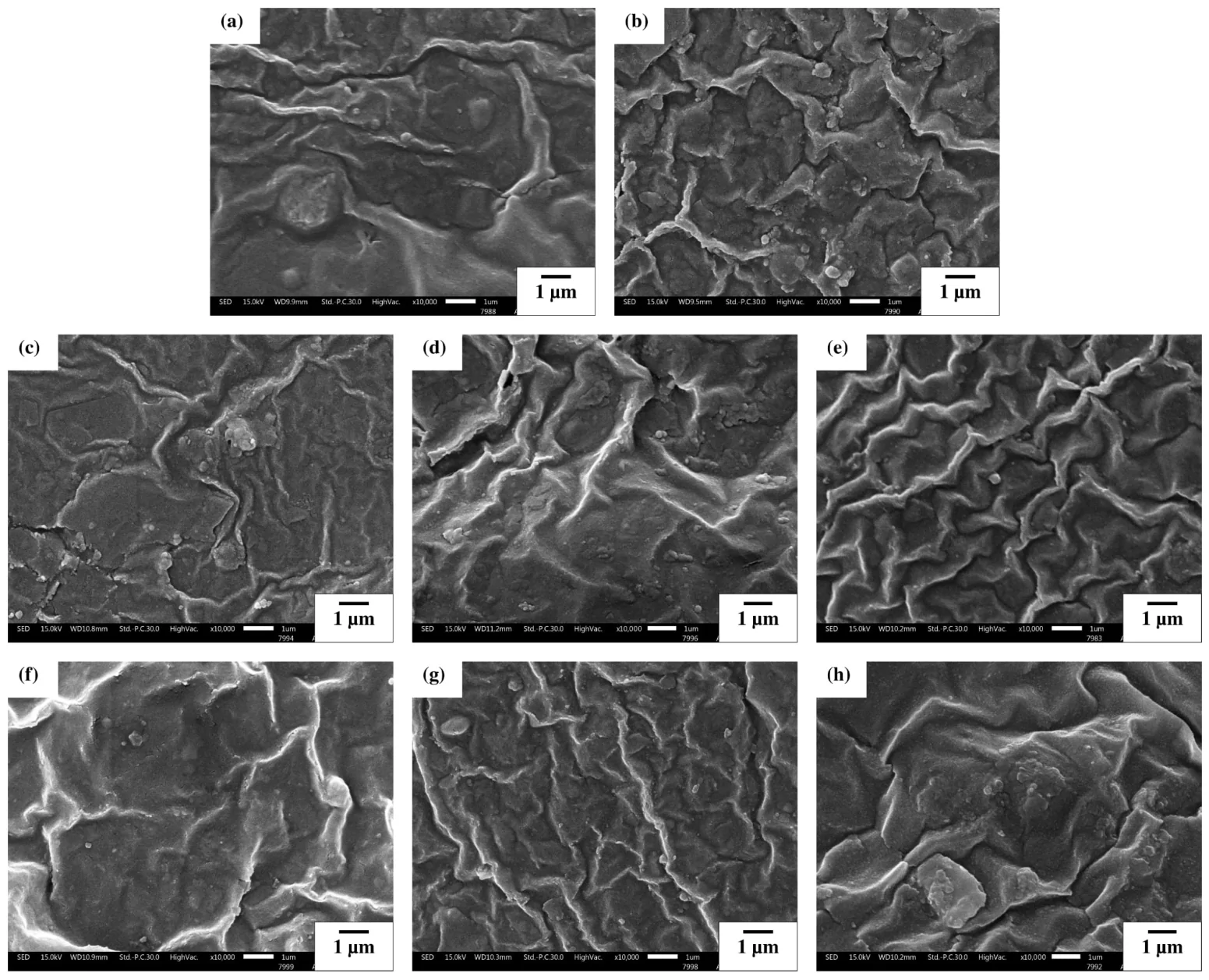
SEM surface images of (**a**) TPS, (**b**) TPS/CMC, (**c**) TPS/Cu, (**d**) TPS/Zn, (**e**) TPS/Ag, (**f**) TPS/CMC/Cu, (**g**) TPS/CMC/Zn and (**h**) TPS/CMC/Ag.

Figure 5 shows the surface images of the sheets and the sparked samples of TPS, TPS/CMC, TPS/Cu, TPS/Zn, TPS/Ag, TPS/CMC/Cu, TPS/CMC/Zn, and TPS/CMC/Ag. The surfaces of TPS and TPS/CMC showed a single-phase miscible structure in the surface images [38,39]. The dispersion of nanometal oxides particles deposited by sparking was observed on the TPS and TPS/CMC surface samples due to the high interfacial adhesion between nanometal oxides particles and TPS surface. The surface of the TPS/CMC samples sparked with nanometal oxides indicated the formation of ionic interactions between nanometal oxides (Cu, Zn, Ag) and the –COOH groups of CMC, consistent with previous reports of ionic interactions between nanometal oxides and –COOH groups [34]. The observed morphological images indicated that the incorporation of CMC influenced the interfacial deposition and distribution of the nanometal particles. The TPS/CMC samples exhibited a relatively continuous distribution of deposited particles without agglomeration of nanometal particles due to the interactions between the deposited metal particles and the polymer surface. This behavior was attributed to the presence of –COOH groups in CMC, which provided additional interaction sites for the metal particles generated during the sparking process. The interfacial interactions enhanced the adhesion of the deposited particles to the TPS/CMC surface and contributed to the formation of localized reinforcing sites. The morphological characteristics were consistent with the enhanced tensile strength observed for the TPS/CMC/nanometal samples, as the improved interfacial interactions contributed to the mechanical reinforcement of the surface-modified TPS/CMC matrix.

**Figure 6:**
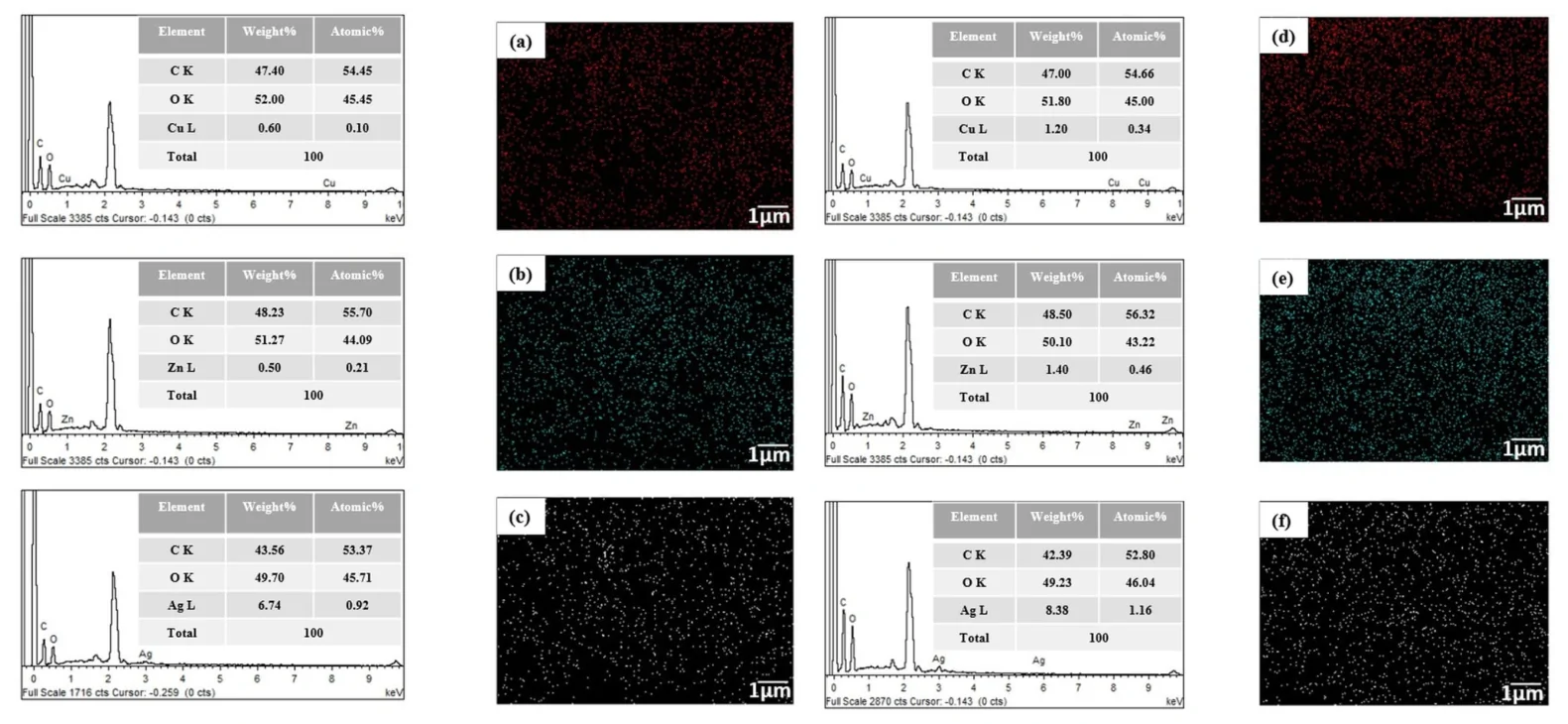
SEM–EDS mapping images: (**a**) Cu mapping of TPS/Cu, (**b**) Zn mapping of TPS/Zn, (**c**) Ag mapping of TPS/Ag, (**d**) Cu mapping of TPS/CMC/Cu, (**e**) Zn mapping of TPS/CMC/Zn and (**f**) Ag mapping of TPS/CMC/Ag.

Figure 6 shows SEM–EDS mode images of the TPS and TPS/CMC surfaces sparked with nanometal oxides (TPS/Cu, TPS/Zn, TPS/Ag, TPS/CMC/Cu, TPS/CMC/Zn, TPS/CMC/Ag). The TPS surfaces sparked with nanometal oxides showed a lower percentage of detected metal elements (Cu, Zn, Ag) compared with the TPS/CMC surface, which attributed to the strong interactions between the –COOH groups of CMC and the nanoparticles. The deposited Cu, Zn, and Ag metals were distributed across the examined polymer surface and were consistent with the surface morphology observed in the SEM images. The uniform distribution of the metal nanoparticles, particularly in the TPS/CMC samples, suggested effective surface modification without the aggregation of metal nanoparticles. The surface modification by sparking contributed to the improvement in tensile strength, which was attributed to the increased ionic interactions and reinforcing effects of the nanoparticles. The embedded nanometal particles acted as localized reinforcing sites, enhancing interfacial adhesion and stress transfer, which improved the mechanical strength of the TPS/CMC samples.

### 3.4. Delta E Color

**Figure 7:**
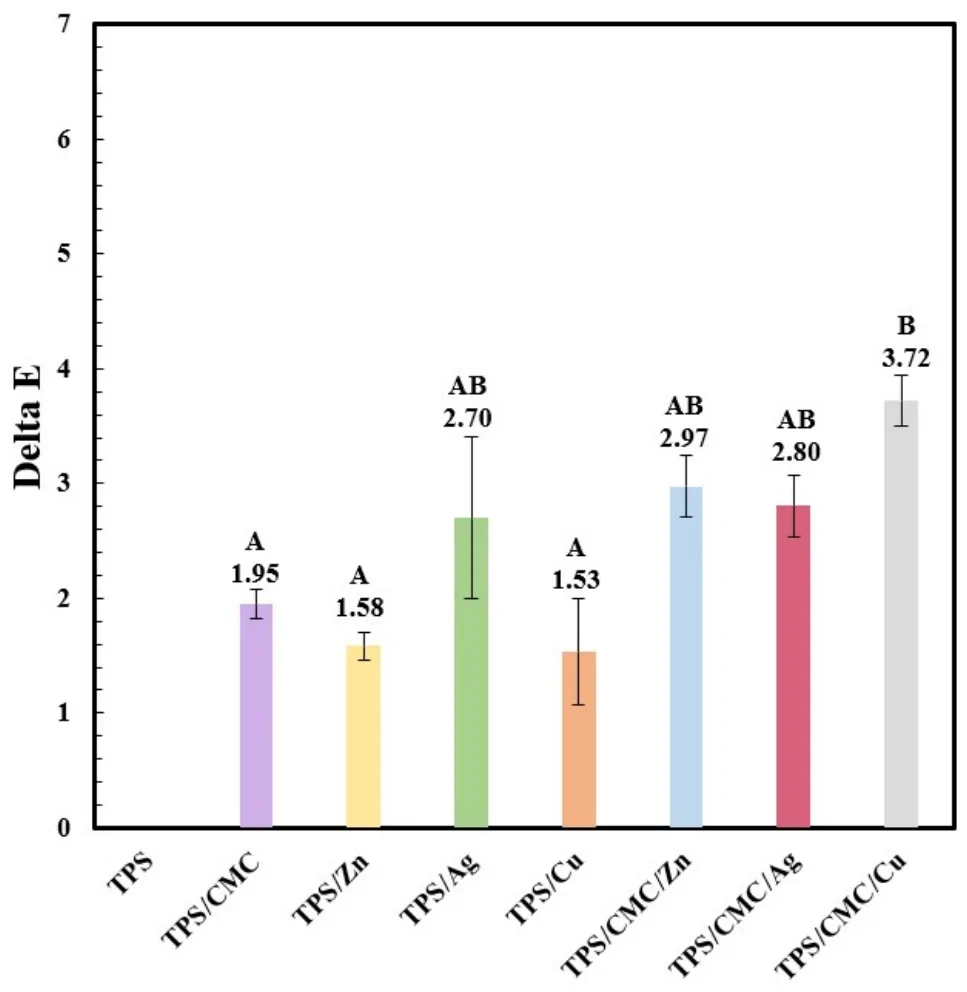
Delta E color of TPS, TPS/CMC, TPS/Zn, TPS/Ag, TPS/Cu, TPS/CMC/Zn, TPS/CMC/Ag and TPS/CMC/Cu. Means with different uppercase letters (A,B) are significantly different (*p* < 0.05).

Figure 7 shows the ΔE values of the TPS, TPS/CMC, TPS/Zn, TPS/Ag, TPS/Cu, TPS/CMC/Zn, TPS/CMC/Ag and TPS/CMC/Cu samples. The L* values of the TPS and TPS/CMC sparked with nanometal oxides (TPS/Zn, TPS/Ag, TPS/Cu, TPS/CMC/Zn, TPS/CMC/Ag, TPS/CMC/Cu) were higher than those of the TPS and TPS/CMC samples, owing to the white color of the samples. The TPS surface sparked with Zn, Ag, and Cu had ΔE colors at 1.58, 2.69, and 1.53, respectively, while the sparked surface of TPS/CMC/Zn, TPS/CMC/Ag, and TPS/CMC/Cu showed ΔE values of 2.97, 2.80 and 3.71, respectively. The sparked samples were more opaque due to the light scattering of nanometal oxide aggregates, while, for sample color values, the L* was higher for the TPS/CMC/Cu sample, owing to the increase in the surface brightness due to the light reflection and scattering caused by the deposition of Cu nanoparticles. The a* values indicated red-toned colors in all samples. The positive a* values indicated a slight shift toward the red color region, which resulted from the changes in light absorption and scattering due to the deposited nanometal particles. Increases in the brightness of polymers, as indicated by higher L* values, following the deposition of nanometal oxides using sparking technology have been reported [16,37]. All samples showed a negative b* value, indicating a yellow color component, together with positive a* values, indicating a red color component, combined with a high L* value (Table 3). The ΔE values of the samples increased after the deposition of nanometal oxides using sparking technology, indicating brightness was increased due to the finely distributed nanometal oxides on the TPS/CMC surface. The difference in the ΔE value between TPS and TPS/CMC after sparking was attributed to the different interfacial interactions between the deposited metal type and the polymer surface.

### 3.5. Contact Angle

**Figure 8:**
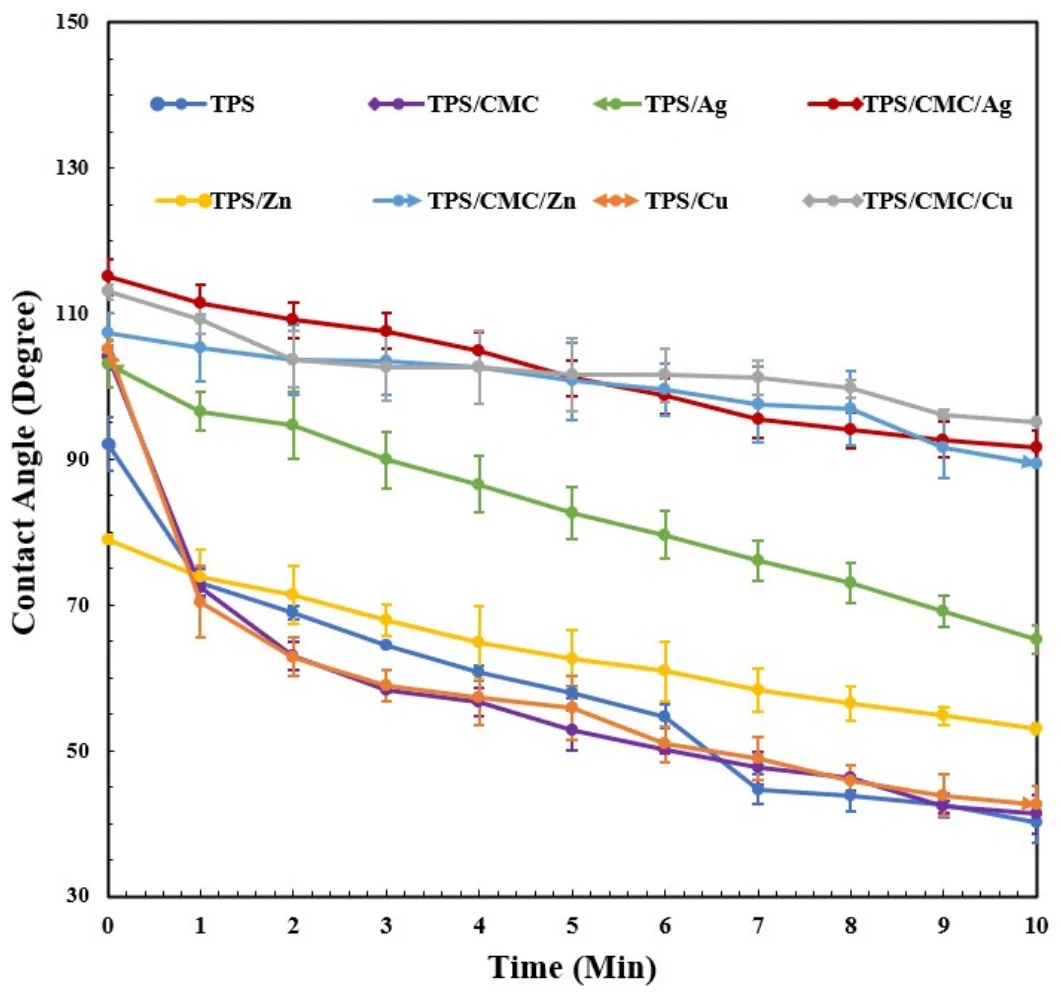
Contact angle of water drops at 0–10 min for TPS, TPS/CMC, TPS/Ag, TPS/CMC/Ag, TPS/Zn, TPS/CMC/Zn, TPS/Cu, and TPS/CMC/Cu samples.

Figure 8 illustrates the contact angle of the TPS, TPS/CMC, TPS/Zn, TPS/Ag, TPS/Cu, TPS/CMC/Zn, TPS/CMC/Ag, and TPS/CMC/Cu samples. The contact angle of the TPS sample decreased from 92.1° to 40.1° over 0–10 min, while that of the TPS/CMC sample decreased from 104° to 41.3°. The TPS/Ag, TPS/Zn, and TPS/Cu samples showed increased contact angles at 10 min, reaching 65.3°, 53.1° and 42.6°, respectively, compared with TPS (40.1°). The deposition of nanometal oxides on the sample surfaces using sparking technology improved surface wettability, as indicated by the increased contact angles, due to the hydrophobic properties and interactions of the nanometal particles [40,41,42]. Interestingly, high contact angle values of 91.6°, 89.3°, and 95.1°at 10 min were observed for TPS/CMC/Ag, TPS/CMC/Zn, and TPS/CMC/Cu, respectively. The water resistance of the TPS/CMC composites with nanometal oxides deposited using sparking technology indicated the formation of ionic interactions between the –COOH groups of CMC and the nanometal oxides; the formation of ionic interactions between nanometal oxides and –COOH groups has been reported [16]. These ionic interactions increased the hydrophobicity and interfacial tension of the samples. A high water contact angle at 10 min after sparking with nanometal oxides was associated with lower surface wettability and higher surface hydrophobicity, indicating the improved surface wettability of the samples. The differences in the contact angles between TPS and TPS/CMC were attributed to differences in the surface functional groups. TPS mainly contains –OH groups, whereas CMC provides –COOH groups that can interact more strongly with the deposited nanometal particles. This stronger interfacial interaction promoted particle retention on the TPS/CMC surface and resulted in a greater change in surface wettability. The differences in the interfacial interactions between the nanometal particles and polymer surfaces also depended on the functional groups present in TPS and TPS/CMC. The –OH groups of TPS and the –COOH groups of CMC exhibited different interactions with Ag, Zn, and Cu, resulting in differences in particle adhesion, surface tension, and wettability.

### 3.6. Thermogravimetric Analysis (TGA)

**Figure 9:**
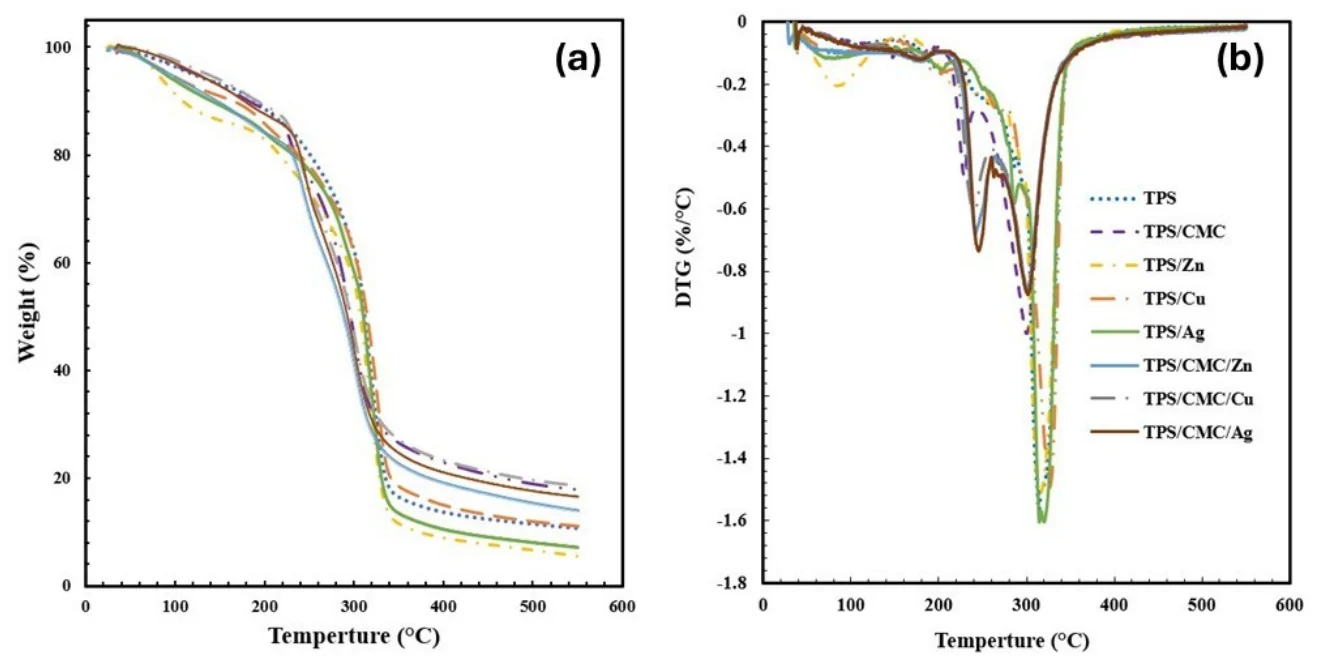
Thermal property measurements: (**a**) TGA thermograms and (**b**) DTG of TPS, TPS/CMC, TPS/Zn, TPS/Ag, TPS/Cu, TPS/CMC/Zn, TPS/CMC/Ag, and TPS/CMC/Cu.

The TGA thermograms of TPS, TPS/CMC, TPS/Zn, TPS/Cu, TPS/Ag, TPS/CMC/Zn, TPS/CMC/Cu, and TPS/CMC/Ag are shown in Figure 9. The first stage at 100 °C was attributed to water evaporation [39], while the second stage at approximately 250–300 °C was related to the thermal decomposition of the polymer components. Lower weight loss was observed for TPS than for TPS/CMC, while the samples sparked with nanometal oxides showed greater weight loss, which was associated with chain degradation [42]. The incorporation of CMC and surface-deposited nanometal influenced the thermal degradation behavior of the TPS matrix. The DTG profiles showed differences in the thermal degradation behavior. The main degradation peaks were observed in the range of approximately 280–330 °C, corresponding to the maximum rate of weight loss of the TPS matrix. The TPS/CMC sample exhibited an additional degradation at a lower temperature (230–245 °C) owing to the degradation of CMC, while CMC shifted the main degradation temperature of the TPS matrix from 320 °C to 300 °C (Table 4). Surface sparking with Ag, Cu, and Zn modified the DTG profiles, resulting in differences in the peak positions and degradation intensity with the nanometal type. The deposited nanometal particles interacted differently with the polymer surface and influenced the thermal decomposition process. The larger residual weight observed for the TPS/CMC and TPS/CMC/nanometal samples at 450 °C was attributed to the inherent thermal decomposition characteristics of the TPS/CMC matrix, rather than the surface-deposited nanometal particles. Interestingly, a similar trend was observed for both TPS and TPS/CMC, with Cu showing a small reduction in residual mass, whereas Ag and particularly Zn resulted in greater reductions. The deposition metal types influenced the thermal degradation of the polymer matrix, with Zn potentially acting as a more effective degradation catalyst than Ag and Cu.

## 4. Conclusions

A new bio-thermoplastic with nanometal sparked on the surface was successfully developed through the preparation of cassava starch and CMC. Nanometal oxides (Ag, Zn, and Cu) were deposited on the surface of TPS and TPS/CMC samples using sparking technology. FTIR suggested interactions between the deposited nanometal particles and the –OH groups of TPS and the –COOH groups of CMC. Sparking the surface of TPS/CMC with nanometal oxides enhanced the mechanical properties, while the morphological and SEM–EDS mapping results confirmed the nanoscale dispersion of the nanometal oxides. The color of the sparked TPS and TPS/CMC samples was changed by Ag, Cu, and Zn due to the changes in brightness and light scattering caused by the nanoparticles. The TGA thermograms showed higher weight loss for TPS/CMC with nanometal particles than for TPS, which was associated with the thermal conductivity and degradation-promoting effect of the nanometal oxide particles. The deposition of nanometal oxides on the TPS/CMC surface using sparking technology effectively increased the contact angle and surface hydrophobicity of the samples. The ionic interactions between the nanometal oxides and the –COOH groups of CMC increased the interfacial adhesion between the TPS and nanometal oxide particles, which improved the properties of the samples. Sparking technology provided an innovative approach for modifying the surface of TPS/CMC by enabling direct nanometal deposition, resulting in improved mechanical strength and surface wettability. Exploring alternative carbohydrate-based polymers and nanomaterials may provide opportunities to further optimize film properties and expand their potential applications.

## Figures and Tables

**Table 1:** The compositions and codes of the prepared samples.

Samples	Composition (*w*/*v*%)		
	Thermoplastic Starch	Carboxymethyl Cellulose	Sparking Nanometal Oxide (30 min)
TPS	100	-	-
TPS/CMC	95	5	-
TPS/Ag	100	-	Ag
TPS/Cu	100	-	Cu
TPS/Zn	100	-	Zn
TPS/CMC/Ag	95	5	Ag
TPS/CMC/Cu	95	5	Cu
TPS/CMC/Zn	95	5	Zn

**Table 2:** FTIR peak position intensity changed with sparking process.

Sample	–COO^−^ Peak (cm^−1^)	Intensity Ratio I_1610_/I_1022_
CMC	1596	-
TPS/CMC	1610	0.257
TPS/CMC/Ag	1610	0.263
TPS/CMC/Cu	1610	0.350
TPS/CMC/Zn	1610	0.293

**Table 3:** L*, a*, and b* values of TPS, TPS/CMC, TPS/Zn, TPS/Ag, TPS/Cu, TPS/CMC/Zn, TPS/CMC/Ag and TPS/CMC/Cu. Means with different uppercase letters, A–E, within the same column are significantly different (*p* < 0.05), according to Duncan’s multiple range test.

Samples	L*	a*	b*
TPS	80.37 ± 0.08 ^D^	3.36 ± 0.06 ^C^	42.65 ± 0.06 ^C^
TPS/CMC	79.73 ± 0.15 ^E^	3.73 ± 0.07 ^B^	44.45 ± 0.14 ^B^
TPS/Zn	81.9 ± 0.12 ^B^	3.34 ± 0.02 ^C^	42.76 ± 0.12 ^C^
TPS/Ag	81.1 ± 0.24 ^C^	4.26 ± 0.27 ^A^	45.05 ± 0.76 ^AB^
TPS/Cu	81.8 ± 0.49 ^B^	3.40 ± 0.02 ^C^	43.02 ± 0.20 ^C^
TPS/CMC/Zn	79.3 ± 0.46 ^E^	3.75 ± 0.07 ^B^	45.37 ± 0.11 ^A^
TPS/CMC/Ag	81.8 ± 0.34 ^B^	4.20 ± 0.19 ^A^	44.92 ± 0.32 ^AB^
TPS/CMC/Cu	83.5 ± 0.43 ^A^	4.07 ± 0.09 ^A^	44.49 ± 0.47 ^B^

**Table 4:** Onset temperature, maximum temperature, and residual value of TPS, TPS/CMC, TPS/Zn, TPS/Ag, TPS/Cu, TPS/CMC/Zn, TPS/CMC/Ag, and TPS/CMC/Cu.

Samples	*T*_onset_ (^°^C)	*T*_max_ (^°^C)	Residue (wt%)
TPS	292.0	310.2	10.69
TPS/CMC	259.5	296.2	14.85
TPS/Zn	290.3	311.5	5.51
TPS/Ag	293.9	310.7	7.13
TPS/Cu	298.6	322.2	8.60
TPS/CMC/Zn	252.5	297.3	13.97
TPS/CMC/Ag	252.5	297.5	13.05
TPS/CMC/Cu	253.6	297.2	14.60

## Data Availability

Data are contained within the article.

## Abbreviations

The following abbreviations are used in this manuscript:

TPS	Thermoplastic starch
CMC	Carboxymethyl cellulose
Zn	Zinc
Ag	Silver
Cu	Copper
